# ‘No idea of time’: Parents report differences in autistic children’s behaviour relating to time in a mixed-methods study

**DOI:** 10.1177/13623613211010014

**Published:** 2021-04-30

**Authors:** Daniel Poole, Emma Gowen, Ellen Poliakoff, Luke A Jones

**Affiliations:** University of Manchester, UK

**Keywords:** autism, questionnaire, thematic analysis, temporal cognition, time perception

## Abstract

An emerging body of research suggests that temporal processing may be disrupted in autistic children, although little is known about behaviours relating to time in daily life. In the present study, 113 parents of autistic and 201 parents of neurotypical children (aged 7–12 years) completed the *It’s About Time* questionnaire and open-ended questions about their child’s behaviour relating to time. The questionnaire scores were lower in the autistic compared with the neurotypical group, suggesting that behaviours are affected. Three key themes were identified using thematic analysis: autistic children had problems with *temporal knowledge*, learning about concepts relating to time, such as how to use the clock and language around time. There were differences in *prospection* with autistic children having more difficulties with how they thought about the future and prepared themselves for upcoming events. The final theme, *monotropism*, described how autistic children viewed their time as precious so they could maximise engagement in their interests. The present study indicates that behaviours relating to time can have a considerable impact on the daily lives of autistic children and their families. Further work exploring the development of temporal cognition in autism would be valuable for targeting effective educational and clinical support.

**Lay abstract:**

Many everyday activities require us to organise our behaviours with respect to time. There is some evidence that autistic children have problems with how they perceive and understand time. However, little is currently known about this, or the ways in which behaviours related to time are impacted in daily life. In this study, 113 parents of autistic children and 201 parents of neurotypical children completed a questionnaire and open-ended questions about their child’s behaviour relating to time. Questionnaire scores were lower in the autistic group compared with neurotypicals, which suggests that behaviours relating to time are affected in autistic children. The open-ended responses further confirmed that the autistic children struggled with time and that this impacted on them and their family. Three key themes were identified. Theme 1: autistic children have problems with learning about concepts relating to time such as telling the time from a clock and using words to describe time (hours, minutes, etc.) appropriately. Theme 2: autistic children think about the future differently. Planning and working under time pressure were described as a problem. Theme 3: autistic children have strong interests which take up a lot of their attention and worrying about having sufficient time to pursue these interests causes anxiety. This research indicates that behaviours related to time can have a considerable impact on the lives of autistic children and that targeted support may be required.

## Introduction

The ability to organise ourselves in time is valuable for many everyday behaviours, including coordinating ourselves with others, planning and waiting for things to happen. The range of complex cognitive processes which sub-serve effective timing (temporal cognition) emerges gradually through development. It has been theorised that there is a rudimentary representation of time in early childhood whereby the status of events in the present moment are represented as ‘completed’ or ‘ongoing’ (McCormack & Hoerl, 2017). Around the age of 5 years, time can be understood independently of events and children begin to conceive of time as linear. Children learn about temporal units (such as days and weeks) through explicit teaching, which is required for the development of a mental ‘time line’ in order to comprehend the relationship between temporal events in the past and future (Friedman, 2003; Hudson & Mayhew, 2011). Diachronic thinking, the ability to understand changes over time, develops from age 6–10 years, as the child’s capacity to think forward and backward in time, and synthesise temporally distinct events into a single construct emerges (Moore et al., 2014; Tryphon & Montangero, 1992). Clock reading is learnt gradually through childhood, many children can read digital clocks by age 6 years and analogue clocks by age 10 years (Friedman & Laycock, 1989). Finally, young children (age <9 years) are less precise when making perceptual estimates of duration compared with older children and adults (Droit-Volet, 2011, 2016; Droit-Volet et al., 2001), which may reflect reduced attention to time, or more variable and/or distorted memory for duration. There is increasing research interest into whether the development of effective timing processes and temporal cognition are disrupted in neurodevelopmental conditions, including attention-deficit/hyperactivity disorder (ADHD; Noreika et al., 2013), dyslexia (Casini et al., 2018; Gooch et al., 2011) and autism. In the present study, we conducted a parental survey of behaviours relating to time in autistic children to better understand how these behaviours are impacted in everyday life.

Autism is characterised by a range of cognitive and behavioural differences encompassing communication and social interaction, sensory processing and motor control (American Psychiatric Association, 2013). It has been theorised that temporal processing deficits are characteristic of autism (the temporal deficit hypothesis, Allman, 2011; Allman & Falter, 2015). According to this theory, issues with temporal processing are far-reaching, encompassing the perception of duration and the relative timing of sensory signals, as well as problems with temporal cognition. As the nature of temporal processing issues in autism has not been well specified, a range of methodological approaches have been used as described below (see Casassus et al., 2019 for a systematic review). Furthermore, basic time perception deficits are suggested to be a mechanism underlying differences in cognition and behaviour in autism. Behaviours such as repetitive movements, or a strong adherence to planning and routine, are proposed to be adaptive, providing temporal boundaries to compensate for diminished precision in the perception of duration. Differences in sensory responsiveness in autism are proposed to result from reduced sensitivity to the relative timing of sensory signals. It is suggested that problems with temporal cognition could impact on planning for future events and in the understanding of cause and effect.

Research using temporal psychophysics has revealed some evidence that autistic children are less precise in the perception and reproduction of duration (Allman et al., 2011; Brenner et al., 2016; Isaksson et al., 2018; Lepistö et al., 2005, 2006; although see Gil et al., 2012; Jones et al., 2017; Wallace & Happé, 2008 for contradictory findings) and the perception of relative timing of stimuli (Kwakye et al., 2011; Stevenson et al., 2014; although see Puts et al., 2014) compared with neurotypical controls. Time-based prospective memory, the ability to remember to complete one’s previous intentions after some duration, is reduced in autistic children (Altgassen et al., 2019; Henry et al., 2014; Williams et al., 2013, 2014). Encoding of prospective memory is believed to be underpinned by the ability to conceptualise oneself performing the given task in the future (Ford et al., 2012). There is evidence suggesting that diachronic thinking is impacted in autism (Boucher et al., 2007). Autistic children produced fewer descriptions of past and future (diachronic tendency), change over time (diachronic transformation) and the ability to think of a succession of states or events over time as a whole (diachronic synthesis). Overall, differences from neurotypical performance have been more commonly observed in the small number of studies which have investigated aspects of temporal cognition, involving the interaction of timing processes with other aspects of cognition such as working memory, whereas research using temporal psychophysics to investigate time perception has generated more mixed findings (see Casassus et al., 2019).

Although more work is required to better understand the cognitive mechanisms underlying timing differences in autism, it is also important to move beyond experimental measures developed to assess performance in neurotypical samples in order to characterise everyday behaviour relating to time in autistic children. Here, we are referring to behaviour where the individual is cognisant of a temporal component. For instance, regarding time management, patience, the appropriate use of temporal concepts and the ways in which thinking about the past and future shapes behaviour in the present moment.

The *It’s About Time* (IAT) questionnaire is a 25-item parent-report scale developed to explore the theory that reduced inhibitory control in ADHD may also impact on timing and time-oriented behaviour in the condition (Barkley et al., 1997). The IAT was designed to assess the child’s sense of time, tendency to talk about time, punctuality and ability to meet work deadlines with lower scores indicating more difficulty in these areas. The parent is asked to respond to questions about their child’s time management abilities (e.g. If your child has a deadline to meet, how often is he or she likely to be ready or prepared for that deadline? How often does your child refer to a watch or clock in planning how much time he or she has left to do something?), future and past thinking (e.g. How often does your child seem to think about their past or use their hindsight before responding in a situation? If your child promises to do something for you at a later time that day, how likely is he or she to remember to do it without being reminded?) and time concept (e.g. In general, compared with other children of their age, how well developed is your child’s sense of time?). IAT scores are reduced in children with ADHD (Quartier et al., 2010) and adults (Riccio et al., 2005) in comparison with individuals without ADHD. To date, there have been just two studies with autistic children which have used the IAT as part of a wider psychophysical timing assessment. Both studies revealed reduced IAT scores in the autistic group (Allman et al., 2011 in a sample of 13 autistic and 12 neurotypical children aged 7–16 years, *d* = 3.53 and Isaksson et al., 2018 in a sample of 17 autistic and 18 neurotypical children aged 8–15 years, *d* = 1.90). The latter study found that IAT scores correlated with performance on a free finger tapping task, whereby autistic children who chose to tap at a faster rate had reduced IAT scores. This may suggest a link between motor timing and everyday behaviours related to time in autism. In a recent qualitative study, autistic adults described interrupted time experiences (Vogel et al., 2019). Participants completed the Time Questionnaire (Vogel et al., 2020) which contained open-ended questions regarding the experience of the passage of time and concepts related to the past, present and future. Participants described a low awareness of the passage of time (a generally ‘bad sense of time’) which could be ameliorated using structure and routine. Routine was also described as improving the experience of the present moment and in reducing anxieties associated with the near future. This study suggests that autistic adults adapt their behaviours to reduce negative experiences arising from problems relating to temporal perception and cognition.

A better understanding of time-based behaviour in autism has a high relevance in an educational and clinical setting. In neurotypicals, effective time management is associated with educational and work-based attainment and improved well-being (Britton & Tesser, 1991; Claessens et al., 2007; Macan et al., 1990). In the present study, we used a mixed-methods approach to better characterise behaviours relating to time in autistic children. First, we measured IAT scores to test whether the previous observation of reduced IAT scores in autistic children in comparison to neurotypicals (Allman et al., 2011; Isaakson et al., 2018) could be replicated in a much larger sample. Second, we used open-ended questions so that parents could describe their child’s behaviour in their own words. This qualitative survey data was analysed using thematic analysis (Braun et al., 2020) to obtain a rich perspective on behaviours related to time beyond the scope of the existing literature.

## Method

Materials, data, analysis code and supplementary materials are available on the Open Science Framework (https://osf.io/6kg9z/).

### Participants

**Figure 1.**
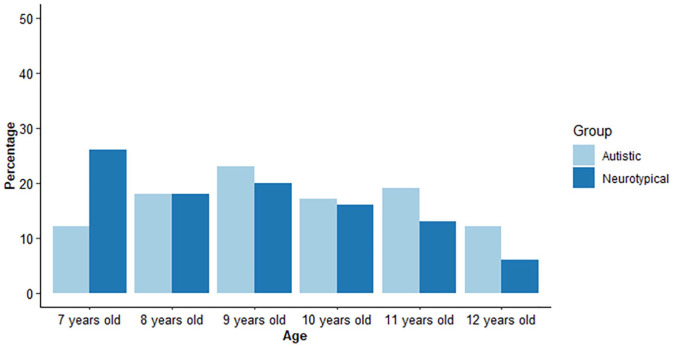
Children’s ages in the autistic (light) and neurotypical (dark blue) groups.

All parents recruited to the study had a child aged 7–12 years, based in the United Kingdom and attending mainstream education. In the United Kingdom, it is a statutory requirement that by age 8–9 years children have been taught to read, tell and write the time from both digital and analogue clocks, and solve problems relating to chronology and language around time (Department for Education, 2013). The distribution of children of different ages in each group is given in Figure 1.

A total of 113 parents of autistic children (7 fathers) completed the questionnaire. Parents of autistic children were recruited via ASD-UK, which is a database of families of autistic people managed by the University of Newcastle and Autistica. Families joining the database had previously provided a letter from a clinician confirming their child’s diagnosis. Parents described their child’s language ability as ‘uses sentences with good grammar’ (*n* = 105), or as ‘uses mostly simple phrases’ (*n* = 8). Parents reported additional diagnosis of ADHD (*n* = 8), dyslexia (*n* = 5), dyspraxia (*n* = 4), sensory processing disorder (*n* = 4), epilepsy (*n* = 3), chromosome disorders (*n* = 3), hypermobility (*n* = 3), anxiety disorder (*n* = 3) and sleep disorder (*n* = 1). Note that co-occurring diagnosis were reported by the parent and were not verified.

In all, 201 parents of neurotypical children (25 fathers) completed the questionnaire. Parents of children in the neurotypical group were recruited via the research teams’ existing contacts, participant recruitment websites, social media, local schools and advertisements at the University of Manchester. An additional exclusion criterion in the neurotypical group was having a first-degree relative with a diagnosis of autism (i.e. sibling or parent). All parents reported their child’s language use as ‘uses sentences with good grammar’. Parents reported diagnosis of dyslexia (*n* = 3), hypermobility (*n* = 2), epilepsy (*n* = 1), sensory processing disorder (*n* = 1), ADHD (*n* = 1) and tic disorder (*n* = 1). Parents in both groups who reported co-occurring diagnosis in their children were included in the analysis. Specific data on socioeconomic status and ethnicity were not obtained for either group.

Before arriving at the final sample size, participants who did not complete the IAT questionnaire were removed (autistic group *n* = 15; neurotypical group *n* = 16). In addition, participants who responded as NA to every item were removed (autistic group *n* = 1; neurotypical group *n* = 1). Finally, a participant in the neurotypical group was removed, who indicated in the open response items that their child had a diagnosis of autism. Ethical approval was obtained from the University of Manchester Ethics Committee. All participants confirmed informed consent online before being directed to the questionnaire. Participants did not receive any payment in compensation for taking part in the study.

### Materials

Participants completed an adapted version of the IAT questionnaire and five open-ended questions. The materials were developed through discussion with the parents of autistic children and autistic adults (via both the Autism@Manchester expert by experience group and the ASD-UK research panel). The scale of the IAT questionnaire was re-worded to *Never*, *Sometimes*, *Often* and *Always* and an NA response was included where an item was not relevant (for instance, where the child was too young to play out alone). There was additional minor rewording of items (details of all changes are included in the supplementary details). Cronbach’s alpha was 0.87 for the autistic group and 0.80 for the neurotypical group.

The open-ended questions were developed in collaboration with the Autism@Manchester expert group. We aimed to develop a small number of questions to generate a rich qualitative dataset regarding behaviours related to time in everyday life (allowing parents to expand on their responses on the IAT in their own words). As such, questions were designed to be relevant to the everyday experiences of autistic and neurotypical children aged 7–12 years. In addition, it was important that the questions were unambiguous and comprehensible to the general public. Qualitative surveys typically require more closed questioning than interviews in order to focus responses without an interviewer present to clarify meaning (Braun et al., 2020). The five-open-ended questions were as follows:

1. Can you think of any occasions where punctuality has affected your child?
2. How does your child prepare so they can do things, such as going to school, on time? Do they (or you) use any particular strategies to help them get ready?
3. The extent to which we are aware of the passage of time can change depending on what we are doing. Does your child ever appear more, or less, aware of the passage of time? If so, what activities have they been engaged in when you have noticed this?
4. To what extent do any differences in the understanding and experience of time impact your child’s life?
5. If you have any other comments about your child’s understanding and experience of time, please add them here.

### Procedure

Participants completed the survey online. Participants completed demographic information, the adapted IAT questionnaire and the open-ended questions on separate pages in that order. Participants were required to provide a response to each item. The mean (standard deviation) time taken to complete the survey was 18 (2) minutes.

### Analysis

Data processing and analysis was conducted using R; 1.5% of responses in the autistic group and 0.8% in the neurotypical group were NA responses. NA responses were replaced with imputed values using multiple correspondence analysis using the *missMDA* package (Josse & Husson, 2016). The proportion of NA responses on each item for each group is included in the supplementary materials. Responses in the imputed dataset were coded numerically (*Never* = 0 to *Always* = 4). The sum of all items for each individual gave their IAT score. A Mann–Whitney U test was used to compare IAT scores between the groups. Cliff’s delta (*d*) and confidence intervals were calculated using the *effSize* package (Torchianio, 2020). We repeated the analysis removing all participants with co-occurring diagnosis as outlined in the participants section, the conclusions remained the same. There was also no effect or interaction with age (these additional analyses are included in the supplementary materials).

A total of 105 parents in the autistic group and 177 in the neurotypical group provided responses on the open-ended questions. Qualitative analysis of responses to the open-ended questions was led by D.P. and managed using the *RQDA* package (Huang, 2018). The autistic group’s responses to open-ended responses were analysed using thematic analysis to provide an in-depth interpretative analysis of the data (Braun & Clarke, 2006). A codebook-type approach (see Braun et al., 2019) incorporating inductive coding was used, whereby a code set was developed and gradually refined through analysis of the entire dataset. After familiarisation with the entire dataset, including note taking on possible relevant themes, the responses from the autistic group were exhaustively coded by D.P. A second pass was then conducted to refine the entire code set with codes being rewritten and similar codes being merged. Codes were identified at both a semantic (summary of the response) and latent level (interpreting the meaning behind the response). D.P. is a neurotypical researcher with many years of experience of working with autistic people; many of the codes relate to concepts from cognitive psychology reflective of his training in this area. D.P. then exhaustively applied the code set to responses from the neurotypical group to provide quantitative summaries to identify aspects of the themes that were unique to the autistic group and which experiences both groups had in common. Descriptions of the codes and quantitative summaries for the autistic and neurotypical group are included on the study OSF page.

The entire coded dataset was then checked independently by L.A.J. and E.G., and where required, codes were relabelled or reworded to improve clarity via discussion. L.A.J. and E.G. are non-autistic researchers with a background in time perception and autism, respectively. D.P. then organised codes from the autistic group into categories linked according to overarching key themes with reference to the dataset. Finally, D.P. returned to the participant responses to confirm the themes which provided a good account of the data from the autistic group and to look for any evidence of these themes in the neurotypical group; this was checked by E.G.

### Community involvement

The study materials were developed in collaboration with autistic adults and the parents of autistic children via the Autism@Manchester expert group and the ASD-UK panel.

## Results

### IAT scores

**Figure 2.**
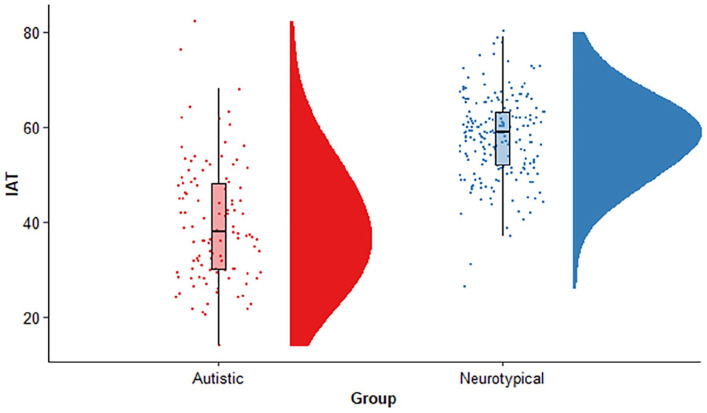
Raincloud plot displaying total score on the adapted *It’s About Time* questionnaire (IAT) for the autistic and neurotypical groups. IAT scores were reduced in the autistic group in comparison to neurotypicals.

IAT scores are displayed in Figure 2. Scores were lower in the autistic group in comparison with neurotypical (U = 2624, *p* < 0.001, −19 [95% CI = −22, −17], *Δ* = 0.77 [95% CI = 0.67, 0.84]).

### Open-ended questions

Parents in the autistic group described problems relating to time as having a considerable impact on the well-being of their children. In contrast, problems relating to time described by parents of neurotypical children were negligible. Some quotes from the answers given by parents in the autistic group illustrate this impact:

*. . .* But out of all those sense issues they don’t impact his life as much as his lack of time sense. It impacts his sleep, it impacts his relationships with others and interferes with his own feeling of well-being.

His lack of concept of time is disabling. He needs support.

It has a huge impact. He is constantly ‘on the back foot’ in life. He gets in trouble for not doing things in time, his time management on tests is non-existent. Finishing tasks in a timely fashion is not happening . . .

**Figure 3.**
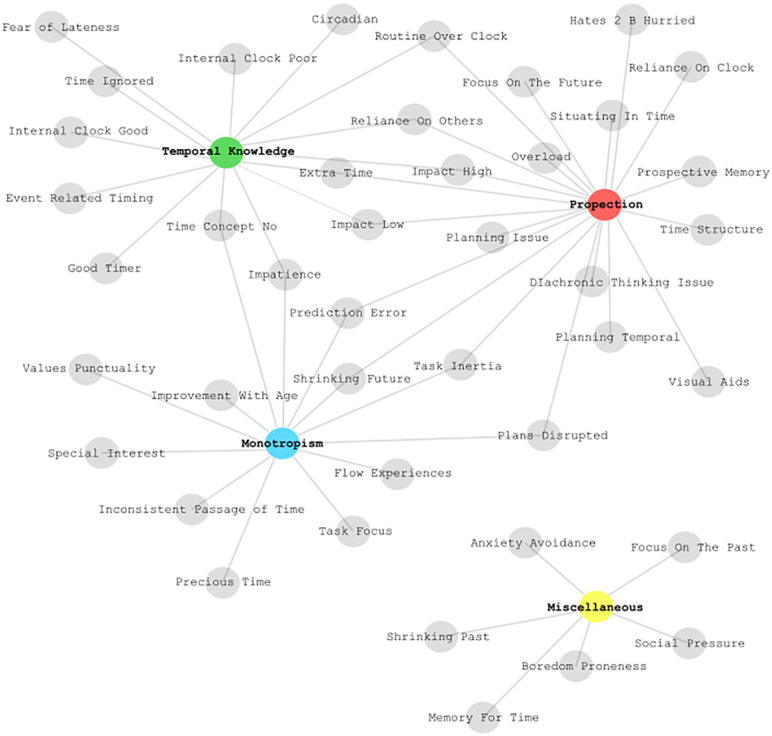
Plot of the organisation of the codes from the open comments made by parents of autistic children into themes. A description of the codes and counts are provided on the study OSF page..

Three central themes were identified: temporal knowledge, prospection and monotropism. The categorisation of the codes which made up each of these themes is represented in Figure 3.

### Theme 1: Temporal Knowledge

Parents described problems their children experienced in learning about concepts related to time and the impact that this had on their everyday experiences. Problems with using clocks were widely described, particularly in reading the time using analogue clocks. The use of language around time was also impacted, such as the appropriate use of temporal units when describing periods of time. There was also a feeling that some children who could use temporal concepts appropriately did not have a strong sense of the durations that they referred to:

She has little understanding and cannot tell the time even at 12 yrs old. Her brain cannot understand the concept.

Playing games with other kids is problematic. If they agree to play his game in 10 minutes but he doesn’t know when 10 minutes is, they land up going home before he gets a turn. Insert rage and feelings of betrayal.

Parents noted an impact on their child’s well-being, particularly increased frustration and confusion arising from diminished temporal knowledge. Children’s awareness of their reduced ability in this regard encouraging an increased focus on, or avoidance of, time. This was particularly reflected in heightened anxieties about punctuality and the social repercussions arising from this:

Lateness is seen as bad to him. He frequently asks if he is late, e.g. every day walking to school or if there is an activity to go to. He does not understand if we are on time or late, so asks very frequently.

She is almost obsessed with time because she doesn’t understand it. It impacts every aspect of her life as she’s constantly worried about being late or having too much to do and not enough time.

Many children relied on regularly occurring events to act as cues to compensate for their diminished understanding about time, for instance, knowing morning is when the sun rises. However, when the timing of these events changed (for instance, with the seasons) this was liable to add to confusion and anxiety.

On the contrary, there were a small number of examples, whereby autistic children were described as having typical, or above average, sense of time for their age. Those who had an excellent understanding of time were motivated to teach themselves how to read the clock and recognise temporal concepts. However, this improved temporal knowledge was not associated with a concomitant improvement in planning abilities (see Theme 2), suggesting understanding clocks and temporal units would not be sufficient to remediate all problems relating to time.

My daughter realised the importance of clocks and time at a very young age. She was determined to understand how to tell the time and effectively taught herself by constantly asking what the time was when looking at a clock or watch.

I actually think he has a hyperacute sense of time and has been able to tell the time from an unusually young age. I don’t know if that means he is good at sensing the passage of time though and he is not good at planning ahead.

Parents in the neurotypical group generally described their children as having good understanding of temporal concepts and any descriptions of weaker temporal knowledge were seen as proportionate to the child’s age.

### Theme 2: Prospection

This theme related to children’s capacity to imagine the future and prepare for future events. This was comprised of two sub-themes that were merged, relating to mental time travel and planning.

Differences in thinking about and conceiving of future states of the world were commonly described by parents of autistic children:

My son has absolutely no idea of time. He thinks his Bday could be tomorrow or expects Christmas in summer etc. If I say, something is happening in 2 weeks, a second later he will ask if that’s tomorrow . . .

When thinking about the future, it may be that the (non-conscious) tendency to anticipate what *may* happen and to flexibility update these predictions based on what has happened previously is impacted in some way (see Lawson et al., 2014). Instead, a detailed, structured model of future states of the world is developed in order to anticipate upcoming activities and events. This was not only when preparing for immediately upcoming events; one child had planned his gap year (year out of education after finishing school aged 18 years). This way of thinking of the future was inflexible, meaning that when events did not map onto the previous expectations it could be a considerable cause of distress:

If an event happens at a different time he says his mind goes into panic, his mind shuts down and he is unable to think about anything until he processes it, but he is deeply unhappy after that and struggles to engage with the activity.

Problems with planning were widely described and had a considerable impact on the child, and their family’s well-being. There were marked problems with remembering previously made plans, even where these were integrated into highly structured routines. The autistic children were also overloaded when presented with multiple tasks involving time pressures:

He sleeps in his school uniform so he does not have to get dressed in the morning. He understands that this is not hygienically preferable however it works for him. He only needs to put on his shoes in the morning.

When starting a task in school and has been set a time limit he will panic and not do any of it because he is so worried about the time limit and not having enough time to complete the set task.

Extensive parental support was used to navigate regular time pressures, such as preparing to get to school and managing homework deadlines. Visual aids, such as timetables, were widely used and could be valuable in supporting the child in managing their time independently. However, reports of the efficacy of visual aids were mixed and, in many instances, parents needed to provide structured support so that their child remembered to use them. Most commonly, parents reported supporting their children through close monitoring and prompting, which was a cause of stress for both child and parent. In addition, parents were concerned that planning was an important life skill that their child would not have an opportunity to develop due to their intervention, but the immediacy of time pressures, such as the demand to be punctual for school, meant there was a lack of opportunity to work on this with their children. Although school created many time pressures which were described as a daily source of stress, the routines and structures of the school week were also seen to be valuable in making each day predictable for the children.

Problems with planning and parental intervention to support this, particularly in relation to getting ready for school, were also commonly described by parents in the NT group. However, the extent and negative impact on both child and parent was clearly considerably less in comparison with the autistic group. Many descriptions referred to specific examples rather than a generalised issue. Problems were described as an inconvenience rather than a major source of stress and anxiety.

### Theme 3: Monotropism

Autism has been characterised by an ‘attentional tunnel’ whereby cognitive resources are allocated towards a closed and relatively narrow range of preferred interests (*monotropism*; Murray et al., 2005). In the context of the present study, children’s behaviours related to time were determined by their interest in a given task.

Autistic children were described as viewing their time as highly precious and time spent on activities unrelated to their interests was seen as wasted. A high value was placed on punctuality in others:

If people are late he then obsesses with how late and what that means to his plans.

He can get anxious if he feels that he is ‘losing time’. Eg he has to do homework and can get worried if he feels that he is missing out on computer time.

Impatience when waiting for preferred activities was widely reported. Children seemed to have heightened attention to time immediately before an activity they were interested in. Indeed, it was reported that frustration associated with waiting for something they wanted to do could overwhelm the child to the extent that their participation in the activity would be affected, or would have to be abandoned.

The ability to focus on a task was modulated by the child’s interests. When completing regular tasks, which they were less interested in, their attention would be very easily drawn elsewhere. When fully immersed in an activity, they could enter a state of flow, seemingly losing awareness of their surroundings and the passage of time. When interrupted, the child would express shock at how long had passed:

If he is waiting to do something he knows the exact number of seconds and gets restless quickly. When absorbed in an activity we have to set a timer as he doesn’t realise the passage of time.

On the contrary, there were also reports that children became extremely conscious of time while engaged in activity. For instance, actively monitoring the time throughout to ensure that they could maximise the opportunity to engage:

He sometimes pauses a tv programme or movie to check how much longer is left when he doesn’t want it to end, so he’s aware of time then, even if he doesn’t know how much time has passed, he’s checking

Time itself also featured as an interest, including pleasure derived from precise and apparently arbitrary time keeping:

He’s quite fascinated by time and how long things take – how long train journeys take, how long it takes to fly from a to b, whether a train we have caught will have reached its destination (if we get off at an earlier station). He can tell the time well and has a clock by his bed.

He loves wearing a watch and timing ‘things’, but cannot work to a deadline.

There were also reports that timing was modulated by the child’s interest in the NT group. This was focused on time seeming to fly by for the child when they were engaged in an activity as a consequence of the child not monitoring the time. The intensity of the interests was diminished in comparison with the autistic group, as well as the variety; comments tended to focus on screen time.

## Discussion

In the present study, we investigated parental reports of behaviour related to time in autistic and neurotypical children. As anticipated, scores on the IAT questionnaire were reduced in the autistic group. We extended previous work by using thematic analysis of parent’s responses to open-ended questions about their children’s behaviour. Responses indicated that issues around behaviours relating to time increased stress and anxiety in their children. Three key themes were identified: *temporal knowledge* identified problems autistic children had with learning about concepts relating to time, including using clocks and language around time appropriately. *Prospection* described differences in how autistic children think about and prepare themselves for the future, encompassing issues related to planning and organising their behaviour with respect to time. Finally, *monotropism* included details of how autistic children oriented their time around activities and events which resonated with their interests. Parents in the neurotypical group described limited issues around behaviours relating to time in their children. These three themes were not reflected in the neurotypical group data.

The present study makes a valuable contribution to the literature investigating time in autism, which has largely been focused on temporal psychophysics (Casassus et al., 2019). We have replicated the previous finding that IAT scores are reduced in an autistic sample with a large effect size (Allman et al., 2011; Isaksson et al., 2018), in a much larger sample than previous work. The qualitative data has provided novel insight into the nature of differences in everyday behaviours relating to time in autistic children in their parent’s own words. By including a neurotypical control group we were able to identify which aspects of the themes were unique to the autistic group and which were comparable to non-autistic children of a similar age. A recent study also used an open-ended questionnaire to better characterise time in autism (Vogel et al., 2019). The authors used a theoretical framework (derived from work with neurotypicals; Vogel et al., 2020) regarding the phenomenological experience of time, which proposes that the experience of time arises from distinct, but interacting, components of the passage of time and time structure (the past, present and future). Content analysis was used in order to empirically validate the proposal that time experiences are interrupted in autism, as a consequence of reduced interaction between the theorised components. In the present study, we used a more inductive, reflexive approach, drawing on parental accounts of their child’s behaviours relating to time. Although the aims, methodological approaches and age group studied differ between these studies, there were notable overlaps in the findings. Similar to the present study, autistic adults described the uncertainty of the future as a cause of anxiety which they managed through precise, but inflexible, planning. Also, the participants described that their interest in an activity determined how they experienced time and flow states were commonly reported. In addition, there were insights into how the participants experience the passage of time in the present moment which were possible to capture as this study used a first-person perspective. In future work, it would be valuable to explore phenomenological experiences of time in autistic children and adolescents.

Some responses to the open-ended questions could be interpreted according to the temporal relevance (TR): temporal uncertainty (TU) theory of attention to time (Zakay, 1992, 2015) According to this theory, the experience of the passage of time is modulated by the interaction between TR and TU. If it is important to a person that something happens on time (e.g. if waiting for a bus for a date) then TR, and attention to time, is increased. When attention to time increases, temporal information is sought, if TU is high (e.g. the bus service is unreliable) then the duration will feel extended and seem to drag by. To synthesise with the current findings, it may be that in many situations for autistic children TR (in relating, or delaying access, to monotopic interests) is high, increasing attention towards time. TU may also be high (as a consequence of diminished temporal knowledge), the increased attention to time with high TU could lead to increases in anxiety and frustration. Empirical studies investigating the nature of TR:TU in autistic children could provide valuable insight into negative effects associate with timing. For instance, comparing judgements of durations relating to or delaying the individual’s interests versus other activities.

The current findings have highlighted the impact of problems relating to time on autistic children’s everyday life. The findings of the present study indicate that many experience severe challenges when processing information and organising their behaviour with respect to time. There are likely to be educational implications, in particular, autistic children may be at a disadvantage whenever completing work under time pressure (such as exams). Flexibility regarding punctuality for school may help reduce anxiety. Targeted support may be required when learning about temporal concepts and in developing a framework for thinking about time and the future. In addition, clinical interventions could be directed towards supporting parents with managing their child’s anxieties around time. However, more work is required to understand the nature of timing differences and the underlying cognitive mechanisms.

The interpretation of the open-ended questions suggests some hypotheses for future neurocognitive research. For instance, whether clock reading abilities relate to visuospatial processing issues, working memory or duration perception differences in autism (or some interaction between these cognitive abilities). The recent dual systems theory of temporal cognition (Hoerl & McCormack, 2018) may provide a useful framework for future investigation in autism. Briefly, this approach proposes that a temporal updating system is present in early infancy which provides a model of the present moment, updated according to past events. A temporal reasoning system develops around the age of 5 years as the child develops an increasingly four-dimensional concept of time, involving flexible changes in perspective between the changing present, past and future and the understanding that all events have a relative location denoted by time. It may be that the maturation of temporal reasoning is delayed in autistic children leading to problems in grasping temporal concepts and thinking about the future. This is currently speculative, but a line of research assessing autistic children’s temporal reasoning skills may be a useful approach in further understanding time in the condition.

### Limitations

First, the IAT is not a validated questionnaire. The focus of items on the IAT is broad and it is not measuring a clearly operationalised construct. Nevertheless, it is valuable in the present study in providing a wide-reaching survey relating to time. As the present study has illustrated the relevance of issues relating to time in autistic children’s lives, psychometrically validated measures of timing would be valuable to assess difficulties and offer effective support. Second, in using written responses there was no opportunity to ask participants to elaborate further on specific points. Follow-up work could use in-depth interviews in a smaller group of parents with children who experience problems relating to time. However, parental report can only provide insight across a limited range of contexts and perception of the child’s behaviour will invariably be shaped by knowledge of the child’s diagnosis (Ringer et al., 2020). Triangulation would be valuable in future work with the perspective of teachers (Tobia et al., 2019), and the children themselves providing a more complete picture of the impact of timing on everyday life. Third, the groups in this study were not IQ matched, so it is not possible to discount differences in intellectual functioning as a mediating factor in the differences in behaviours related to time reported here.

## Conclusion

In the present study, quantitative and qualitative data from the parents of autistic children has suggested that there are problems relating to time in the condition, which have a considerable impact on daily life. Parents described problems relating to learning about temporal concepts and thinking about the future. In addition, behaviours relating to time were modulated by the child’s interests. This work suggests that further work exploring temporal cognition in autism would be valuable in providing effective support.

## References

[bibr1-13623613211010014] Allman M. J. (2011). Deficits in temporal processing associated with autistic disorder. Frontiers in Integrative Neuroscience, 5(3), Article 2. 10.3389/fnint.2011.00002 21472033 PMC3068294

[bibr2-13623613211010014] Allman M. J. DeLeon I. G. Wearden J. H. (2011). Psychophysical assessment of timing in individuals with autism. American Journal on Intellectual and Developmental Disabilities, 116(2), 165–178. 10.1352/1944-7558-116.2.165 21381951 PMC4822529

[bibr3-13623613211010014] Allman M. J. Falter C. M. (2015). Abnormal timing and time perception in autism spectrum disorder? A review of the evidence. In Vatkis A. Allman M. J. (Eds.), Time distortions in mind: Temporal processing in clinical populations (pp. 37–56). Brill Academic.

[bibr4-13623613211010014] Altgassen M. Sheppard D. P. Hendriks M. P. H. (2019). Do importance instructions improve time-based prospective remembering in autism spectrum conditions? Research in Developmental Disabilities, 90(1), 1–13. 10.1016/j.ridd.2019.04.008 31015072

[bibr5-13623613211010014] American Psychiatric Association. (2013). Diagnostic and statistical manual of mental disorders (5th ed.). American Psychiatric Publishing.

[bibr6-13623613211010014] Barkley R. A. Koplowitz S. Anderson T. McMurray M. B. (1997). Sense of time in children with ADHD: Effects of duration, distraction, and stimulant medication. Journal of the International Neuropsychological Society, 3(4), 359–369. 10.1017/s1355617797003597 9260445

[bibr7-13623613211010014] Boucher J. Pons F. Lind S. Williams D. (2007). Temporal cognition in children with autistic spectrum disorders: Tests of diachronic thinking. Journal of Autism and Developmental Disorders, 37(8), 1413–1429. 10.1007/s10803-006-0285-9 17171540

[bibr8-13623613211010014] Braun V. Clarke V. (2006). Using thematic analysis in psychology. Qualitative Research in Psychology, 3(2), 77–101.

[bibr9-13623613211010014] Braun V. Clarke V. Boulton E. Davey L. McEvoy C. (2020). The online survey as a qualitative research tool. International Journal of Social Research Methodology. Advance online publication. 10.1080/13645579.2020.1805550

[bibr10-13623613211010014] Braun V. Clarke V. Hayfield N. Terry N. (2019). Thematic analysis. In Liamputtong P. (Ed.), Handbook of research methods in health and social sciences (pp. 1–18). Springer.

[bibr11-13623613211010014] Brenner L. A. Shih V. H. Colich N. L. Sugar C. A. Bearden C. E. Dapretto M. (2016). Time reproduction performance is associated with age and working memory in high-functioning youth with autism spectrum disorder. Autism Research, 8(1), 29–37. 10.1002/aur.1401.Time PMC4312276 25078724

[bibr12-13623613211010014] Britton B. K. Tesser A. (1991). Effects of time-management practices on college grades. Journal of Educational Psychology, 83(3), 405–410. 10.1037/0022-0663.83.3.405

[bibr13-13623613211010014] Casassus M. Poliakoff E. Gowen E. Poole D. Jones L. A. (2019). Time perception and autistic spectrum condition: A systematic review. Autism Research, 12(10), 1440–1462. 10.1002/aur.2170 31336032 PMC6852160

[bibr14-13623613211010014] Casini L. Pech-Georgel C. Ziegler J. C. (2018). It’s about time: Revisiting temporal processing deficits in dyslexia. Developmental Science, 21(2), Article e12530. 10.1111/desc.12530 28239921

[bibr15-13623613211010014] Claessens B. J. C. Van Eerde W. Rutte C. G. Roe R. A. (2007). A review of the time management literature. Personnel Review, 36(2), 255–276. 10.1108/00483480710726136

[bibr16-13623613211010014] Department for Education. (2013). The national curriculum in England: Complete framework for key stages 1 to 4. 10.1080/09571739185200191

[bibr17-13623613211010014] Droit-Volet S. (2011). Child and time. In Giagkou M. Vatakis A. Papadelis G. Cummins F. Esposito A. (Eds.), Multidisciplinary aspects of time and time perception (pp. 151–173). Spring-Verlag.

[bibr18-13623613211010014] Droit-Volet S. Clement A. Wearden J. (2001). Temporal generalisation in 3- to 8- year old children. Journal of Experimental Child Psychology, 80, 271–280. 11583526 10.1006/jecp.2001.2629

[bibr19-13623613211010014] Droit-Volet S. (2016). Development of time. Current Opinion in Behavioral Sciences, 8, 102–109. 10.1016/j.cobeha.2016.02.003

[bibr20-13623613211010014] Ford R. M. Driscoll T. Shum D. Macaulay C. E. (2012). Executive and theory-of-mind contributions to event-based prospective memory in children: Exploring the self-projection hypothesis. Journal of Experimental Child Psychology, 111(3), 468–489. 10.1016/j.jecp.2011.10.006 22169353

[bibr21-13623613211010014] Friedman W. (2003). The development of children’ s knowledge of the times of future events. Child Development, 71(4), 913–932. 10.1111/1467-8624.00199 11016556

[bibr22-13623613211010014] Friedman W. Laycock F. (1989). Children’s analogue and digital clock knowledge. Child Development, 60(2), 357–371.

[bibr23-13623613211010014] Gil S. Chambres P. Hyvert C. Fanget M. Droit-Volet S. (2012). Children with autism spectrum disorders have ‘the working raw material’ for time perception. PLOS ONE, 7(11), Article e49116. 10.1371/journal.pone.0049116 PMC3504053 23185299

[bibr24-13623613211010014] Gooch D. Snowling M. Hulme C. (2011). Time perception, phonological skills and executive function in children with dyslexia and/or ADHD symptoms. Journal of Child Psychology and Psychiatry and Allied Disciplines, 52(2), 195–203. 10.1111/j.1469-7610.2010.02312.x PMC3412207 20860755

[bibr25-13623613211010014] Henry J. D. Terrett G. Altgassen M. Raponi-Saunders S. Ballhausen N. Schnitzspahn K. M. Rendell P. G. (2014). A Virtual Week study of prospective memory function in autism spectrum disorders. Journal of Experimental Child Psychology, 127, 110–125. 10.1016/j.jecp.2014.01.011 24679459

[bibr26-13623613211010014] Hoerl C. McCormack T. (2018). Thinking in and about time: A dual systems perspective on temporal cognition. Behavioral and Brain Sciences, 25, Article 42. 10.1017/S0140525X18002157 30251619

[bibr27-13623613211010014] Huang R. (2018). RQDA: R-based qualitative data analysis. R package version 0.3.1.

[bibr28-13623613211010014] Hudson J. A. Mayhew E. M. Y. (2011). Children’s temporal judgments for autobiographical past and future events. Cognitive Development, 26(4), 331–342. 10.1016/j.cogdev.2011.09.005

[bibr29-13623613211010014] Isaksson S. Salomäki S. Tuominen J. Arstila V. Falter-Wagner C. M. Noreika V. (2018). Is there a generalized timing impairment in Autism Spectrum Disorders across time scales and paradigms? Journal of Psychiatric Research, 99, 111–121. 10.1016/j.jpsychires.2018.01.017 29438910

[bibr30-13623613211010014] Jones C. R. G. Lambrechts A. Gaigg S. B. (2017). Using time perception to explore implicit sensitivity to emotional stimuli in autism spectrum disorder. Journal of Autism and Developmental Disorders, 47(7), 2054–2066. 10.1007/s10803-017-3120-6 28429189 PMC5487748

[bibr31-13623613211010014] Josse J. Husson F. (2016). MissMDA:a package for handling missing values in multivariate data analysis. Journal of Statistical Software, 70, 1–31.

[bibr32-13623613211010014] Kwakye L. D. Foss-Feig J. H. Cascio C. J. Stone W. L. Wallace M. T. (2011). Altered auditory and multisensory temporal processing in autism spectrum disorders. Frontiers in Integrative Neuroscience, 4(1), Article 129. 10.3389/fnint.2010.00129 PMC3024004 21258617

[bibr33-13623613211010014] Lawson R. P. Rees G. Friston K. J. (2014). An aberrant precision account of autism. Frontiers in Human Neuroscience, 8, Article 302. 10.3389/fnhum.2014.00302 PMC4030191 24860482

[bibr34-13623613211010014] Lepistö T. Kujala T. Vanhala R. Alku P. Huotilainen M. Näätänen R. (2005). The discrimination of and orienting to speech and non-speech sounds in children with autism. Brain Research, 1066(1–2), 147–157. 10.1016/j.brainres.2005.10.052 16325159

[bibr35-13623613211010014] Lepistö T. Silokallio S. Nieminen-von Wendt T. Alku P. Näätänen R. Kujala T. (2006). Auditory perception and attention as reflected by the brain event-related potentials in children with Asperger syndrome. Clinical Neurophysiology, 117(10), 2161–2171. 10.1016/j.clinph.2006.06.709 16890012

[bibr36-13623613211010014] Macan T. H. Shahani C. Dipboye R. L. Phillips A. P. (1990). College students’ time management: Correlations with academic performance and stress. Journal of Educational Psychology, 82(4), 760–768. 10.1037/0022-0663.82.4.760

[bibr37-13623613211010014] McCormack T. Hoerl C. (2017). The development of temporal concepts: Learning to locate events in time. Timing and Time Perception, 5(3–4), 297–327. 10.1163/22134468-00002094

[bibr38-13623613211010014] Moore B. D. Brooks P. J. Rabin L. A. (2014). Comparison of diachronic thinking and event ordering in 5- to 10-year-old children. International Journal of Behavioral Development, 38(3), 282–292. 10.1177/0165025414520806

[bibr39-13623613211010014] Murray D. Lesser M. Lawson W. (2005). Attention, monotropism and the diagnostic criteria for autism. Autism, 9(2), 139–156. 15857859 10.1177/1362361305051398

[bibr40-13623613211010014] Noreika V. Falter C. M. Rubia K. (2013). Timing deficits in attention-deficit/hyperactivity disorder (ADHD): Evidence from neurocognitive and neuroimaging studies. Neuropsychologia, 51(2), 235–266. 10.1016/j.neuropsychologia.2012.09.036 23022430

[bibr41-13623613211010014] Puts N. A. Wodka E. L. Tommerdahl M. Mostofsky S. H. Edden R. A. (2014). Impaired tactile processing in children with autism spectrum disorder. Journal of Neurophysiology, 111, 1803–1811. 10.1152/jn.00890.2013 24523518 PMC4044368

[bibr42-13623613211010014] Quartier V. Zimmermann G. Nashat S. (2010). Sense of time in children with attention-deficit/hyperactivity disorder (ADHD). Swiss Journal of Psychology, 69(1), 7–14. 10.1024/1421-0185/a000002

[bibr43-13623613211010014] Riccio C. A. Wolfe M. Davis B. Romine C. George C. Lee D. (2005). Attention deficit hyperactivity disorder: Manifestation in adulthood. Archives of Clinical Neuropsychology, 20(2), 249–269. 10.1016/j.acn.2004.07.005 15708734

[bibr44-13623613211010014] Ringer N. Wilder J. Gustavsson A. (2020). Managing children with challenging behaviours. Parents ‘ meaning-making processes in relation to their children’s ADHD diagnosis. International Journal of Disability, Development and Education, 67, 376–392. 10.1080/1034912X.2019.1596228

[bibr45-13623613211010014] Stevenson R. A. Siemann J. K. Schneider B. C. Eberly H. E. Woynaroski T. G. Camarata S. M. Wallace M. T. (2014). Multisensory temporal integration in autism spectrum disorders. The Journal of Neuroscience, 34(3), 691–697. 10.1523/JNEUROSCI.3615-13.2014 24431427 PMC3891950

[bibr46-13623613211010014] Tobia V. Bonifacci P. Bernabini L. Marzocchi G. M. (2019). Teachers, not parents, are able to predict time processing skills in preschoolers. British Journal of Developmental Psychology, 37, 519–534. 10.1111/bjdp.12294 31264234

[bibr47-13623613211010014] Torchianio M. (2020). Effsize. R package version 0.7.8.

[bibr48-13623613211010014] Tryphon A. Montangero J. (1992). The development of diachronic thinking in children: Children’s ideas about changes in drawing skills. International Journal of Behavioral Development, 15(3), 411–424.

[bibr49-13623613211010014] Vogel D. H. V. Falter-Wagner C. M. Schoofs T. Krämer K. Kupke C. Vogeley K. (2019). Interrupted time experience in autism spectrum disorder: Empirical evidence from content analysis. Journal of Autism and Developmental Disorders, 49(1), 22–33. 10.1007/s10803-018-3771-y 30284137

[bibr50-13623613211010014] Vogel D. H. V. Falter-Wagner C. M. Schoofs T. Krämer K. Kupke C. Vogeley K. (2020). Flow and structure of time experience – Concept, empirical validation and implications for psychopathology. Phenomenology and the Cognitive Sciences, 19(2), 235–258. 10.1007/s11097-018-9573-z

[bibr51-13623613211010014] Wallace G. L. Happé F. (2008). Time perception in autism spectrum disorders. Research in Autism Spectrum Disorders, 2(3), 447–455. 10.1016/j.rasd.2007.09.005

[bibr52-13623613211010014] Williams D. M. Boucher J. Lind S. Jarrold C. (2013). Time-based and event-based prospective memory in autism spectrum disorder: The roles of executive function and theory of mind, and time-estimation. Journal of Autism and Developmental Disorders, 43(7), 1555–1567. 10.1007/s10803-012-1703-9 23179340

[bibr53-13623613211010014] Williams D. M. Jarrold C. Grainger C. Lind S. E. (2014). Diminished time-based, but undiminished event-based, prospective memory among intellectually high-functioning adults with autism spectrum disorder: Relation to working memory ability. Neuropsychology, 28(1), 30–42. 10.1037/neu0000008 24128041 PMC3906801

[bibr54-13623613211010014] Zakay D. (1992). On prospective time estimation, temporal relevance and temporal uncertainty. In Macar F. (Ed.), Time, action and cognition (pp. 109–121). Springer.

[bibr55-13623613211010014] Zakay D. (2015). The temporal-relevance temporal-uncertainity model of prospective duration judgement. Consciousness & Cognition, 38, 182–190. 26524983 10.1016/j.concog.2015.10.006

